# Remineralisation of enamel erosive lesions by daily-use fluoride treatments: network meta-analysis of an in situ study set

**DOI:** 10.1007/s00784-024-06107-1

**Published:** 2024-12-26

**Authors:** Jonathan Creeth, Gary Smith, Billy Franks, Anderson Hara, Domenick Zero

**Affiliations:** 1 Haleon PLC, St George’s Avenue, Weybridge, KT13 0DE UK; 2 Oral Health Research Institute, Indiana University School of Dentistry, 415 Lansing Street, Indianapolis, IN 46202-2876 USA

**Keywords:** Toothpaste, Mouthwash, Remineralisation, Demineralisation, Clinical study, Erosive toothwear

## Abstract

**Objectives:**

Daily-use fluoride products are first-line protection against enamel wear from dietary-acid exposure (DAE). This study aimed to understand effects of fluoride concentration, fluoride salt, product form and ingredients in daily-use products on remineralisation and demineralisation, via network meta-analysis (NMA) of 14 studies using one well-established in-situ model. Remineralisation (surface-microhardness recovery, SHMR) after treatment, and protection against subsequent demineralisation (acid-resistance ratio, ARR) were measured.

**Materials and methods:**

Healthy participants, wearing intra-oral palatal appliances holding enamel specimens eroded with standardised DAE, used test products once. Enamel hardness was assessed (Knoop microhardness probe) pre-DAE; post-DAE; after 4 h intra-oral remineralisation; and after post-remineralisation DAE. NMA was performed using a mixed-models approach on subject-level data to estimate and compare means.

**Results:**

There was a dose-response for fluoride ion in toothpastes (0-1426ppm F; *p* < 0.001 for SMHR and ARR). One toothpaste (silica-based, 1150ppm F as NaF) showed a benefit for SMHR versus placebo [mean(standard error)]: 8.8%(0.6%) (33.0% vs. 24.2%; *p* < 0.001); for ARR: 0.27(0.03) (0.43 vs. 0.15; *p* < 0.001; 9 mutual studies). Use of fluoride mouthwash after fluoride toothpaste increased SMHR [2.4%(1.1%); *p* = 0.043; 3 studies]; the effect on ARR [0.08(0.05)] was not significant (*p* = 0.164). Negative effects of polyvalent metal ions and polyphosphates on SMHR (*p* < 0.05) were observed.

**Conclusions:**

NMA proved effective in discriminating between fluoride-based treatments in this in-situ study, highlighting the importance of fluoride ion to enamel protection and showing formulation ingredients can affect its performance.

**Clinical Relevance:**

Daily-use fluoride products can protect enamel against dietary acids, but careful formulation is required for optimal performance.

## Introduction

Erosive tooth wear is a loss of tooth structure due to acids in the diet (or regurgitated from the stomach) directly demineralising tooth surfaces, making them softer and more prone to wear by physical contact [1]. Dental plaque is not involved. The condition is progressive and has become widespread; due, it is believed, to the higher level of acidic foods and drinks in modern diets, and seems to be still increasing [2–6].

Measuring clinical progression of erosive tooth wear is technically difficult, as enamel surface changes are, in most cases, slow: generally, it takes years for visible changes to occur. Furthermore, these changes occur on irregular, curved natural surfaces, without fixed reference points, which makes quantifying enamel surface loss between two time points challenging. So, any difference between treatments being compared in a study will likely be extremely small on a typical clinical time-scale of days to months. Hence, few prospective studies of treatment effects on erosive tooth wear have been attempted [7].

While control of the diet is the logical primary route to reduce risk of erosive tooth wear (from extrinsic acid exposure) [8, 9], fluoride in daily-use oral care products – in practice, toothpastes and mouthwashes - is believed to protect against dietary acids [8, 10–13] as part of an oral hygiene routine. Significant questions remain however, about the role of fluoride in protecting against erosive tooth wear, and more evidence is required concerning the effect of (i) concentration of fluoride in the product, (ii) the vehicle used (mouthwash versus, or in addition to, toothpaste), and (iii) the bioactivity of fluoride in the product.

The third point arises because oral care products, particularly toothpastes, are complex formulations, providing a multiplicity of separate oral care benefits besides those from fluoride. From a remineralisation/demineralisation perspective, most toothpaste ingredients can be considered ‘bystanders’, unlikely to affect these processes. However, several commonly used ingredients employed to achieve separate oral care benefits can potentially influence enamel remineralisation and/or demineralisation. Some may reduce fluoride performance by reducing fluoride bioavailability in the formulation. More importantly in modern formulations, they may inhibit fluoride’s actions on enamel, via their own affinity for enamel surfaces. On the other hand, this same enamel affinity may make enamel more acid resistant than fluoride alone, adding to its protective effect.

In summary, therefore, the effect of different toothpaste ingredients on the known protective effect of fluoride on early erosive lesions is unclear. To elucidate questions regarding the effect on erosive tooth wear processes of fluoride concentration, treatment vehicle, or other formulation ingredients in a fluoride toothpaste requires clinical studies. But because in vivo clinical studies that could clearly differentiate between fluoride treatments are not yet practicable, in situ models - using enamel specimens mounted in intra-oral appliances - have become the most practical, cost-effective, and rapid method of determining efficacy of treatments in practice. In situ approaches allow use of perfectly flat enamel specimens with highly standardized initial mineralisation status that can be examined on the bench after treatment, using sensitive micro-analytical techniques not possible in the mouth.

There are two broad types of in situ methods: those that measure enamel surface loss and those that measure mineralisation changes (without actual surface loss). These two approaches have given sometimes-contrasting results (e.g [14, 15].

In total, our research group has conducted 14 in situ studies over about 15 years focusing on mineralization change, with only minor variations in protocol, testing a total of 22 treatments. The individual studies were analysed for their intended objective. Now, with this wealth of data, further aspects and comparisons can be explored that would not have been possible from the single studies. Of particular interest was the fact that one treatment (Pronamel original toothpaste, 1150 ppm F as sodium fluoride) had been tested versus a fluoride-free placebo in 9 separate studies, facilitating an accurate assessment of the effect of fluoride and the reproducibility of the model.

To achieve this assessment, a network meta-analysis (NMA) technique was employed. NMA is an increasingly popular way to compare treatments using both direct evidence (from within the same study) and indirect evidence (linear estimates based on combining direct evidence [e.g. A vs. B = (A vs. C) – (B vs. C)]. It can combine data sets to make overall estimates of treatment effect sizes.

The present NMA aimed to determine, from the series of in situ erosion studies conducted by our group, the relative effects of the different treatments, in the conditions of the protocol, which were consistent across the studies. The analysis did not aim to compare, *across in situ or clinical methods*, whether one treatment is more or less effective than another in protecting against dental erosion: it was not a systematic review. A similar NMA approach was employed previously to achieve a cross-study comparison of fluoride treatments using an in situ caries model [16].

A network analysis approach was therefore employed, applying a separate primary NMA mixed model to examine treatment effects in this in situ study set. Subject-level data was available from all constituent studies.

The aims of the study were therefore to understand, across a set of 14 in situ studies using a consistent methodology, the effect on early enamel erosive lesion remineralisation and demineralisation, of:

- fluoride ion concentration and fluoride source in a toothpaste.
- fluoridated mouthwash compared to, and in addition to, fluoride toothpaste.
- the effects of different toothpaste formulations, in terms of the potential impact of their ingredients on remineralisation-demineralisation processes: particularly polyphosphates, polyvalent metal ions, and surfactants.

The consistency of the in situ model was also assessed via the reproducibility of the comparison between the fluoride-free placebo and a specific toothpaste formulation with 1150 ppm F performed in nine of the studies in the set.

## Materials and methods

### In situ methodology and ethical approval

The included studies were single-centre, randomized, examiner- and analyst-blind, multi-period, multi-treatment crossover studies. All studies except one were performed at the Oral Health Research Institute at the Indiana University School of Dentistry. These studies obtained ethical approval from the Indiana University Institutional Review Board. The remaining study (code name RH02535, see Table 1) was performed at Intertek Clinical Research Services Ltd, Ellesmere Port, UK. This study obtained ethical approval from the Manchester Consumer Healthcare Research Ethics Committee. All studies were designed according to CONSORT guidelines and were conducted in accordance with the Declaration of Helsinki, the International Conference on Harmonisation of Technical Requirements for Registration of Pharmaceuticals for Human Use and local laws and regulations. All studies were sponsored and funded by Haleon (known as GSK Consumer Healthcare at the time the studies were performed).

**Table 1:** Details of in situ clinical studies included in the network meta-analysis. Details of the specific treatments tested in these studies are provided in Table 2

#	Citation	Internal Study code^1^	Year completed	*N*	brushing duration (s)^2^	In situ remineralisation times	Study Treatment Coding (see Table 2)				
1.	Zero 2006	SMA0501	2009	15	25 + 60	4 h only	AQF000	PNP020	PNP110	CCP110	-
2.	Barlow 2009 (study A)	Z2560412	2009	58	25 + 60	4 h only	PNP000	PNP140	CCP140	-	-
3.	Barlow 2009 (study B)	Z2560490	2009	56	25 + 60	4 h only	PNP000	PNP110	CCP110	CPH110	
4.	Barlow 2009 (study C)	Z3590585	2009	56	25 + 60	4 h only	PNP000	PNP110^3^	PNP110^3^	CSM110	-
5.	Maggio 2010	Z3480664	2009	36	25 + 60	4 h only	PNP000	PNP140	PNP004	PNP144	CRB140
6.	Data on file	Z3480561	2010	55	25 + 60	4 h only	PNP000	PNP110	PNP112	CCP110	-
7.	Data on file	Z6961036	2011	56	25 + 60	4 h only	PNP000	PNP110	PNG110^3^	PNG110^3^	
8.	Data on file	Z6961385	2012	56	25 + 60	2 h, 4 h, 8 h	PNP000	PNP140	PNG140^3^	PNG140^3^	-
9.	Creeth 2015	RH01299	2012	62	25 + 60	4 h only	AQF000	AQF020	AQF110	AQF140	-
10.	Nehme, 2015	RH01925	2014	24	25 + 60	4 h only	PNP000	PNP002	PNP110	PNP112^4^	PNP112^4^
11.	Nehme, 2019	RH02534	2015	50	25 + 60	5,10,15,30min1h,2 h,4 h	PNP000	PNP110	-	-	-
12.	Data on file	RH02535	2015	24	25 + 60	4 h only	PNP000	PNP110	PNP140		-
13.	Creeth 2018	203,111	2015	62	25 + 95	2 h, 4 h, 8 h	PNP000	PNP110	C3D110	PPH110	-
14.	Creeth 2020	208,166	2018	62	25 + 95	2 h, 4 h	PNP000	PIR110	CPS110	-	-

**Table 2:** Coding, description and designation of products, in terms of their content of ingredients with known potential to impact enamel remineralisation-demineralisation processes. The treatments were each given a 6-character code to characterise the formulation(s) used and the details of the fluoride applied. The first 3 characters describe the treatment base formulation (abbreviated brand and product name). Character 4 and 5 describe the fluoride concentration of the toothpaste used: 00 = no added fluoride, 02 = 250ppm F, 11 = between 1000–1150 ppm F, 14 = 1400-1450ppm F. Character 6 describes the fluoride concentration of the mouthwash, when used: 0 = 0ppm, or no use of mouthwash; 2 = 225ppm; 4 = 450ppm). In the text, the treatments are generally referred to by their ‘designation’: i.e. in terms of the presence or absence of fluoride and other ingredients with potential to impact enamel remineralisation or demineralisation

Treatment code	Product identity & description	Fluoride concentration (ppm) & source	Fluoride Dose (µg)	Ingredients potentially impacting remin-demin processes	Designation of treatment ‘chassis’
AQF000	Aquafresh Fresh & Minty non-fluoride placebo toothpaste	No added fluoride	0	SLS^1^	Non-F/SLS
AQF020	Aquafresh Fresh & Minty low-fluoride control toothpaste	250 ppm F as NaF	375	SLS	F/SLS
AQF110	Aquafresh Cavity Protection toothpaste (US)^2^	1100ppm F as NaF	1725	SLS	F/SLS
AQF140	Aquafresh Fresh & Minty toothpaste (EU)^2^	1450 ppm F as NaF	2139	SLS	F/SLS
PNP000	Pronamel Daily Protection non-fluoride placebo toothpaste	No added fluoride	0	-	Non-F/non-SLS^3^
PNP110	Pronamel Daily Protection toothpaste (US)	1150ppm F as NaF	1725	-	F/non-SLS
PNP140	Pronamel Daily Protection toothpaste (EU)	1450 ppm F as NaF	2139	-	F/non-SLS
PNG110	Pronamel Iso-Active Foam toothpaste (US)	1150ppm F as NaF	1725	gel-to-foam formulation	F/non-SLS/GTF
PNG140	Pronamel Iso-Active Foam toothpaste (EU)	1450 ppm F as NaF	2139	gel-to-foam formulation	F/non-SLS/GTF
PPH110	Pronamel Daily Protection toothpaste with 0.85% sodium phytate	1150ppm F as NaF	1725	short-chain polyphosphate (phytate)	F/non-SLS/PP
PIR110	Pronamel Intensive Enamel Repair toothpaste (US)	1150ppm F as NaF	1725	sodium lactate, PVM/MA polymer	F/non-SLS/Lac-PVMMA
CCP110	Crest Cavity Protection toothpaste (US)	1100ppm F as NaF	1650	SLS, orthophosphate, carbomer	F/SLS/PO4/Carb
CCP140	Blend-a-Med toothpaste (EU)^5^	1450 ppm F as NaF	2175	SLS, orthophosphate, carbomer	F/SLS/PO4/Carb
C3D110	Crest 3D White toothpaste (US) Luxe Glamorous White toothpaste	1100ppm F as NaF	1650	SLS, short-chain polyphosphate (pyrophosphate^4^)	F/SLS/PP
CPH110	Crest Pro-Health toothpaste (US) (low-aqueous, sodium hexametaphosphate formula)	1100ppm F as SnF_2_	1650	Stannous ion, zinc ion, SLS, long-chain polyphosphate^6^, sodium hexametaphosphate	F/Sn-Zn/SLS/LPP
CPS110	Crest Pro-Health Smooth toothpaste (US) (high-aqueous, non-polyphosphate formula)	1100ppm F as SnF_2_	1650	Stannous ion, zinc ion, SLS	F/Sn-Zn/SLS
CSM110	Colgate Sensitive Multi-Protection toothpaste (US)	1100ppm F as MFP	1650	Zinc ion, SLS, citrate	MFP/SLS/Zn/Cit
CRB140	Colgate Cavity Protection toothpaste (EU)	1450ppm F (1000ppm as MFP^7^ & 450ppm as NaF)	2175	SLS	MFP/SLS
PNP002	Pronamel Daily Protection non-fluoride placebo toothpaste followed by Pronamel mouthwash (US)	Toothpaste: No added F Mouthwash: 225 ppm F as NaF	2250	-	F-rinse
PNP004	Pronamel Daily Protection non-fluoride placebo toothpaste followed by Pronamel mouthwash (EU)	Toothpaste: No added F; Mouthwash: 450 ppm F as NaF	4500	-	F-rinse
PNP112	Pronamel Daily Protection toothpaste (US) followed by Pronamel mouthwash (US)	Toothpaste: 1150 ppm F as NaF; Mouthwash: 225 ppm F as NaF	3975 (1725 + 2250)	-	F/non-SLS + F-rinse
PNP144	Pronamel Daily Protection toothpaste (EU) followed by Pronamel mouthwash (EU)	Toothpaste: 1450 ppm F as NaF; Mouthwash: 450 ppm F as NaF	6639 (2139 + 4500)	-	F/non-SLS + F-rinse

(1) ‘SLS’=sodium lauryl sulphate, an anionic surfactant; (2) the formulation chassis of Aquafresh Fresh & Minty and Cavity Protection are essentially identical; (3) ‘non-SLS’: instead of SLS, tegobetaine (a zwitterionic surfactant), was used; (4) Pyrophosphate: sodium and/or potassium pyrophosphate; (5) assumed to be a high-fluoride variant of Crest Cavity Protection (US): the ingredients lists are essentially identical; (6) LPP = sodium hexametaphosphate, a long-chain polyphosphate; (7) MFP = sodium monofluorophosphate

### Experimental design

The design of the in situ method, including inclusion & exclusion criteria, is described in detail in earlier publications using this method [17, 18]. A brief summary is provided here.

For the Oral Health Research Institute studies, participants were recruited from an existing panel of individuals who had been pre-fitted with a maxillary palatal appliance (see details below). All were from the Indianapolis, USA area where community water contains approximately 0.75 ppm fluoride. Eligible participants were aged at least 18 years and in good general and oral health. For the Intertek study, performed in a non-fluoridated community water area of the North-West of England, participants were recruited by the site and a palatal appliance was made for each eligible subject post-screening.

Each palatal appliance carried up to 8 bovine enamel specimens. Prior to insertion of specimens into the appliances, surface microhardness (SMH) of each (sound) specimen was measured before and after exposure to an ex vivo dietary acid erosive challenge (these were the baseline (‘B’) and pre-treatment acid challenge (‘E1’) measures respectively). The specimens were sterilised then inserted into the appliance.

Participants attended site for a screening visit, during which they gave written informed consent to take part in the study. Their demographics, medical history, and prior medications were recorded. Oral hard and soft tissue assessments were performed, followed by saliva flow-rate assessment. They then returned for a single visit on each treatment occasion. Treatment visits were separated by at least 3 days with at least a 2 day ‘washout’ period using a non-fluoridated toothpaste immediately prior to the visit.

Subjects wore the appliance for approximately 5 min to allow early pellicle formation on the enamel specimens. They then brushed the occlusal surfaces of their teeth in their normal manner for 25 s with 1.5 g of the test toothpaste, and swished the resulting slurry around their mouths for either 60 s (studies 1–12) or 95 s (studies 13 & 14) to ensure good contact of the toothpaste slurry with the enamel specimens, followed by rinsing for 10s with 15 mL water. For mouthwash treatments, subjects rinsed with 15 mL of product for 60 s.

After a four-hour post-brushing period during which participants wore their appliance to allow saliva-mediated remineralisation, the appliance was removed and specimens extracted for analysis (in certain studies, specimens were also removed at other time-points, but results for these are not included here).

The surface hardness of the specimens was remeasured: this was the post-treatment-induced remineralisation measure (‘R’). A second *ex situ* erosive challenge (identical to the first) was then performed, and the surface hardness measured a final time: this was the post-treatment acid-challenge measure (‘E2’). Enamel fluoride uptake was also measured in certain studies, but the restricted availability of data meant this measure was not included in the NMA.

### Outcome measures

The key experimental measures analysed in the NMA were (i) the post-treatment intra-oral remineralisation, measured as surface microhardness recovery (SMHR) [18]; (ii) the post-remineralisation acid resistance, relative to the pre-treatment acid resistance, measured as the acid resistance ratio (ARR) [19]; and (iii) the overall enamel erosion resistance after a cycle of remineralisation and demineralisation, the relative enamel erosion resistance (RER, also called the net enamel erosion resistance (NER) in some manuscripts) [20]. These measures are derived from the microhardness values obtained during the progression of the testing (B, E1, R and E2) as follows:

1 $$SMHR\;=\;\lbrack(E1-R)/(E1-B)\rbrack\;\ast\;100$$

2 $$\;RER\;=\;\lbrack(E1-E2)\;/\;(E1-B)\rbrack\;\ast\;100$$

3 $$ARR\;=\;1-\lbrack(E2-R)/(E1-B)\rbrack$$

This study focuses on SMHR, to understand treatment effects on *remineralisation* after a dietary acid demineralisation challenge, and also ARR, to understand effects on *demineralisation inhibition* that the treatment-induced remineralisation provides. The third measure, the RER, is actually a relatively straightforward combination of SMHR and ARR without other variables: RER = SMHR + (ARR*100) – 100.

### Studies included in the analysis

Table 1 lists the studies included in the NMA. This represents all in situ remineralisation enamel erosion studies conducted by our research group.

### Product treatments and important ingredients

Product treatments were each allocated a different 6 character code for analysis, according to a convention in which three letters characterised the treatment identity, and three numbers characterised the toothpaste fluoride concentration applied, and the fluoride concentration of any mouthwash used (coding described in full and listed in Table 2).

The formulations were further designated according to their labelled ingredients potentially relevant to fluoride performance. For the purposes of the product treatment comparisons in this analysis, several assumptions were made: (i) the ‘ingredients potentially impacting remin-demin processes’ were the only toothpaste constituents that might affect remineralisation or demineralisation; (ii) SnF2 is assumed to become fully ionised salt during use, so can be considered a source of free fluoride ion - and therefore an identical form of fluoride to that from NaF; (iii) the longer product exposure time in the last two studies [15, 21] − 95 sec swishing vs. 60 sec in earlier studies - did not impact the analysis meaningfully; (iv) for the fluoride dose-response analysis, the F/SLS (SLS is sodium laurylsulphate, formally sodium dodecylsulfate) and F/non-SLS data was combined for each fluoride concentration to increase the power of the analysis (this was supported by the modest effect of SLS observed). The two placebos, SLS and non-SLS, were assumed to perform identically.

The products and product combinations tested in the studies included in this analysis are listed with their assigned codes and designations by potentially impactful ingredients in Table 2.

## Statistical analysis

### Primary and secondary efficacy variables

For each parameter (SMHR, ARR and RER), a separate primary NMA mixed model was applied to the subject period level 4-hour time-point data with product code as a fixed effect and independent random effects for study (overall intercept and for product code effects), period (nested within-study) and subject (nested within-study). Kenward Rogers degrees of freedom was applied.

The estimates of interest (along with SEs, 95% CIs and two-sided p-values) were calculated using a linear combination of the relevant individual product code least-squared means (LSMs) from the primary NMA model.

The linear effect per 100ppm of fluoride (SLS and non-SLS products combined) was estimated as the difference between the average of the SLS and non-SLS product LSMs from the primary NMA model at 0ppm and 1426ppm divided by 14.26. To test for a non-linear fluoride effect, an exploratory mixed model analysis was performed using only data from the SLS and non-SLS product with a covariate (as a continuous variable) for fluoride level (0, 250, 1150 or 1426) and fluoride level squared, and random effects for study (overall intercept and fluoride effect) and subject nested within-study. A lack of evidence of a non-linear effect provided support for the estimated linear effect per 100ppm as described above. If there was evidence of a non-linear effect, such estimates of the linear effect had limited interpretability.

## Results

**Fig. 1:**
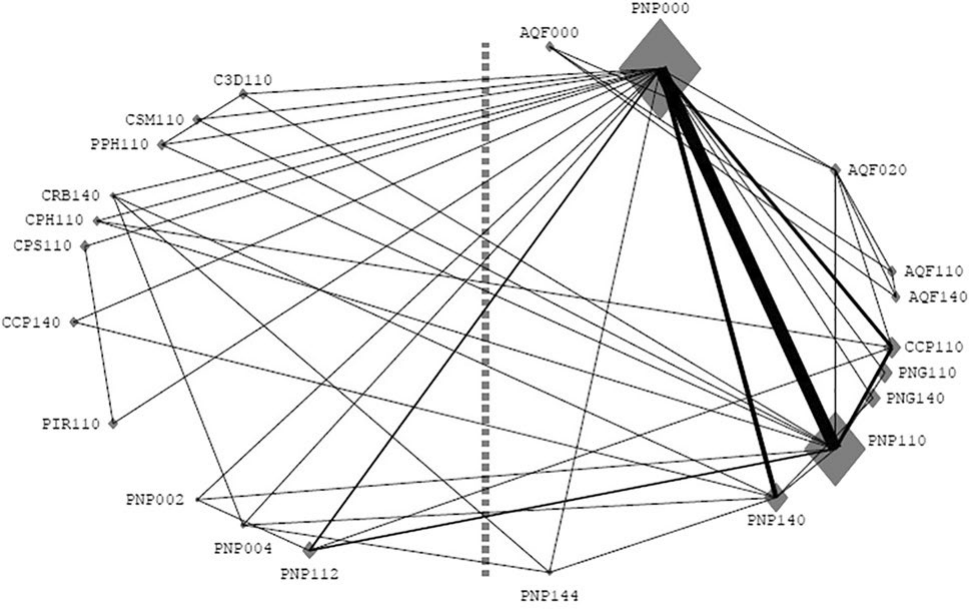
Network Meta-Analysis diagram. The size of symbol represents number of results for the product code across all studies. The thickness of line represents number of subjects with results for both product codes within the same study. Positioning is determined by first and second principal component of SMHR/RER/ARR means. Lower-placed products have higher overall SMHR/RER/ARR values. Symbols to the left or right of the dashed line relate to the consistency or inconsistency of SMHR/RER/ARR, (left: less consistent, right: more consistent) but are not ordered

A schematic representation of the treatment comparisons made, with how many times those comparisons were performed directly in the same study, is presented in Fig. 1.

### Overall pattern of results

The mean indent length for each treatment at each stage of the procedure is shown in Table 3.

**Table 3:** Summary of indent measures for each treatment at each stage of the procedure (adjusted mean *±* standard error). B = Baseline (sound enamel surfaces prior to experiment); E1 = Eroded stage 1 (enamel surfaces from B, after demineralisation, prior to treatment); R = Remineralised enamel surface (enamel surfaces from E1, after product treatment and 4 h of intra-oral remineralisation); E2 = Eroded stage 2 (enamel surfaces from R, after post-treatment demineralisation)

Treatment	# studies	# period-level results at 4 h	B	E1	*R*	E2
AQF000	1	60	43.5 (0.2)	58.4 (0.5)	67.9 (1.7)	54.8 (0.5)
AQF020	2	76	43.6 (0.2)	58.1 (0.4)	64.1 (1.2)	53.9 (0.5)
AQF110	1	61	43.5 (0.2)	58.1 (0.5)	62.7 (1.7)	53.4 (0.5)
AQF140	1	61	43.3 (0.2)	58.1 (0.5)	62.7 (1.7)	53.0 (0.5)
PNP000	13	598	43.7 (0.1)	58.5 (0.3)	67.1 (0.7)	54.8 (0.3)
PNP110	9	443	43.7 (0.1)	58.5 (0.3)	61.8 (0.7)	53.5 (0.3)
PNP140	4	169	43.6 (0.1)	58.4 (0.3)	61.8 (0.9)	53.1 (0.4)
PNG110	1	112	43.7 (0.1)	58.5 (0.3)	62.3 (1.3)	53.5 (0.4)
PNG140	1	109	43.7 (0.1)	58.7 (0.3)	62.1 (1.3)	53.6 (0.4)
PPH110	1	62	43.8 (0.1)	58.7 (0.3)	65.6 (1.3)	55.1 (0.4)
PIR110	1	62	43.6 (0.1)	58.6 (0.3)	62.1 (1.4)	53.6 (0.4)
CCP110*	3	120	43.7 (0.1)	58.6 (0.3)	62.9 (0.9)	53.9 (0.4)
CCP140**	1	57	43.6 (0.1)	58.2 (0.3)	62.4 (1.3)	53.8 (0.4)
C3D110*	1	60	43.8 (0.1)	58.4 (0.3)	65.6 (1.3)	55.3 (0.4)
CPH110*	1	51	43.7 (0.1)	58.6 (0.3)	63.5 (1.3)	55.8 (0.4)
CPS110*	1	62	43.7 (0.1)	58.6 (0.3)	63.9 (1.4)	54.8 (0.4)
CSM110*	1	55	43.7 (0.1)	58.3 (0.3)	65.7 (1.3)	55.3 (0.4)
CRB140**	1	36	43.6 (0.2)	58.0 (0.4)	64.1 (1.4)	54.2 (0.4)
PNP002	1	22	43.7 (0.2)	58.3 (0.4)	60.4 (1.3)	53.6 (0.5)
PNP004	1	36	43.7 (0.2)	58.3 (0.4)	60.5 (1.4)	53.3 (0.4)
PNP112	2	99	43.7 (0.1)	58.6 (0.3)	60.4 (1.0)	53.3 (0.4)
PNP144	1	36	43.6 (0.2)	58.4 (0.4)	60.4 (1.4)	52.6 (0.4)

* assumes formulated at 1100ppm F for Crest & Colgate toothpastes; Aquafresh & Pronamel ‘110’ toothpastes contained 1150ppm F

** assumes toothpaste formulated at 1450ppm F for Blend-a-Med (CCP) & Colgate toothpastes; Aquafresh & Pronamel ‘140’ toothpastes contained 1426ppm F

**Fig. 2:**
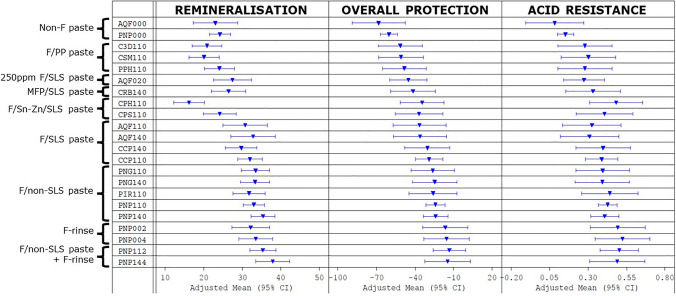
Plot of the NMA results showing adjusted means and 95% confidence intervals for each treatment. Lower-placed products have higher overall SMHR/RER/ARR values

The analysis of remineralisation (measured as %SMHR), demineralisation inhibition (measured as ARR), and overall enamel protection (measured as %RER) for each treatment are summarised in Fig. 2. The treatments are ranked by the first principal component score, across the parameter adjusted means, representing an index of overall performance across the three measures, so that the first and last rows are indicative of the overall lowest and highest performing treatments, respectively.

This plot shows evidence of a hierarchy of performance according to fluoride level, vehicle, and the type of ingredients present in the formulation (for the toothpastes). However, care is required with interpretation of the rank order, as differences may not be statistically significant.

There were a large number of comparisons possible between the twenty-two treatments evaluated. Comparisons of particular interest were prespecified and are shown in Table 4, which also provides statistical analysis of treatment differences.

**Table 4:** Pre-specified comparisons of performance of treatments against the various measures of the study from the network meta-analysis

Product Designation comparison	Treatment 1	Treatment 2	Remineralisation (%SMHR)			Overall Enamel Protection (%RER)			Demineralisation inhibition (ARR)		
			Difference (1–2)	Standard error	*p*-value	Difference (1–2)	Standard error	*p*-value	Difference (1–2)	Standard error	*p*-value
F/non-SLS vs. F/SLS/PP	PNP110	C3D110	12.1	1.4	**< **0.001	27.4	8.0	0.004	0.15	0.09	0.099
F/non-SLS vs. F/non-SLS/PP	PNP110	PPH110	9.0	1.4	**< **0.001	24.2	8.0	0.009	0.15	0.08	0.099
F/non-SLS vs. F/Sn-Zn/SLS	PNP110	CPS110	8.8	1.7	0.001	12.8	9.2	0.181	0.02	0.09	0.831
F/non-SLS s F/non-SLS/ Lac-PVMMA	PNP110	PIR110	1.2	1.7	0.497	1.8	9.2	0.846	−0.01	0.09	0.883
F/non-SLS vs. non-SLS	PNP110	PNP000	8.8	0.6	**< **0.001	36.2	3.1	**< **0.001	0.27	0.03	**< **0.001
F/non-SLS vs. F/Sn-Zn/LPP	PNP110	CPH110	16.8	1.5	**< **0.001	10.3	7.8	0.209	−0.06	0.08	0.510
F/non-SLS vs. MFP/SLS/Zn/Cit	PNP110	CSM110	12.9	1.4	**< **0.001	26.8	8.0	0.005	0.13	0.09	0.159
F/non-SLS vs. MFP/SLS	PNP140	CRB140	8.9	1.9	< 0.001	17.7	8.4	0.051	0.08	0.09	0.406
F/non-SLS vs. F/SLS/PO4/Carb	PNP110	CCP110	1.0	1.0	0.346	5.0	5.0	0.329	0.04	0.05	0.467
F/SLS vs. F/SLS/PP	AQF110	C3D110	9.9	3.1	0.004	15.0	12.8	0.256	0.05	0.12	0.715
F/SLS vs. F/Sn-Zn/SLS	AQF110	CPS110	6.6	3.2	0.053	0.4	13.4	0.976	−0.08	0.13	0.528
F/SLS vs. F/Sn-Zn/SLS/LPP	AQF110	CPH110	14.6	3.1	**< **0.001	−2.1	12.6	0.869	−0.16	0.12	0.212
F/SLS vs. MFP/SLS	AQF140	CRB140	6.4	3.3	0.065	5.5	13.1	0.675	−0.02	0.13	0.854
F/non-SLS/Lac-PVMMA vs. F/Sn-Zn/SLS/LPP	PIR110	CPH110	15.6	2.2	**< **0.001	8.5	11.7	0.478	−0.04	0.12	0.729
F/non-SLS/Lac-PVMMA vs. F/SLS/PP	PIR110	C3D110	10.9	2.2	0.001	25.6	11.8	0.046	0.16	0.12	0.199
F/non-SLS/Lac-PVMMA vs. F/SLS/PO4/Carb	PIR110	CCP110	−0.2	1.9	0.919	3.2	10.0	0.754	0.05	0.10	0.608
Paste alone vs. Paste + Rinse (across F concentrations)	PNP110 + PNP140	PNP112 + PNP144	−2.4	1.1	0.043	−10.1	5.1	0.067	−0.08	0.05	0.164
F/non-SLS vs. F/SLS/PO4/Carb (across F concentrations)	PNP110 + PNP140	CCP110 + CCP140	3.3	0.9	0.006	5.7	4.8	0.253	0.02	0.05	0.637
F/non-SLS vs. F/SLS (across F concentrations)	PNP110 + PNP140	AQF110 + AQF140	2.4	2.7	0.382	12.3	9.3	0.198	0.10	0.08	0.234
F/SLS vs. F/SLS/PO4/Carb (across F concentrations)	AQF110 + AQF140	CCP110 + CCP140	1.0	2.8	0.734	−6.6	10.0	0.517	−0.08	0.09	0.414

### Effects of fluoride

#### Fluoride effects at 1150 ppm

**Fig. 3:**
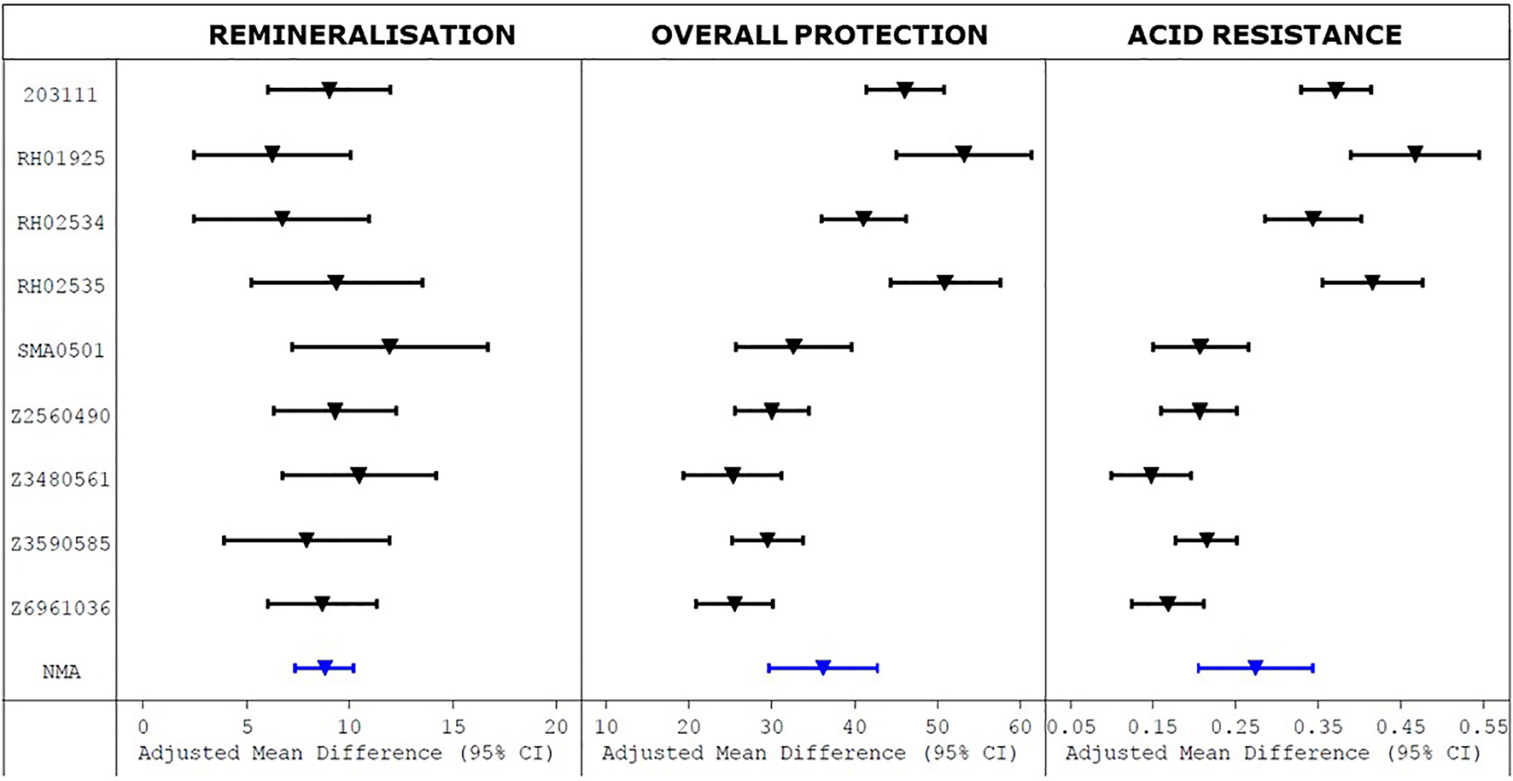
Forest plot of the results of the NMA specifically for the difference between the F/non-SLS (1150 ppm F) and non-SLS toothpastes, showing adjusted means and 95% confidence intervals for the fluoride effect on the study measures

The NMA estimate of the mean effect of 1150 ppm F as NaF (in the F/non-SLS toothpaste) compared to non-SLS fluoride-free placebo (i.e. PNP110 vs. PNP000; nine of the fourteen studies assessed both products) was compared to the individual study comparisons between these two products to calculate an overall mean effect and help assess the consistency and reproducibility of the full NMA model result (Fig. 3).

The mean (standard error) increases from addition of the 1150 ppm F as NaF were 8.8% (0.6%) for remineralisation (%SMHR), 0.27 (0.03) for demineralisation inhibition (ARR), and 36.2% (3.1%) for overall enamel protection (%RER) (all *p* < 0.001; see Table 4).

In terms of reproducibility across studies, the mean absolute remineralisation values for the F/non-SLS toothpaste (PNP110) varied from 25 to 40%; the increments versus non-fluoride control ranged from 6 to 12%. The mean absolute demineralisation inhibition values varied from 0.36 to 0.49; the increments observed versus non-fluoride control ranged from 0.15 to 0.47.

#### Fluoride concentration dose-response

**Fig. 4:**
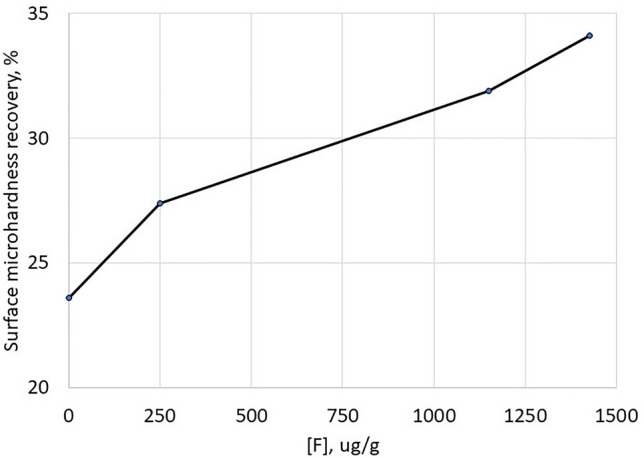
Promotion of remineralisation (measured by surface microhardness recovery) as a function of fluoride concentration in the toothpaste treatment (combined F/non-SLS and F/SLS treatments)

Figure 4 shows the degree of enamel remineralisation as a function of free fluoride concentration (0–1426 ppm F as NaF) in a toothpaste. The mean (95% CI) increase in SMHR per 100ppm increase in fluoride level was 0.7% (0.6–0.9%), *p* < 0.001. The additional exploratory NMA model also did not indicate a non-linear fluoride dose-response for remineralisation (*p* = 0.427).

**Fig. 5:**
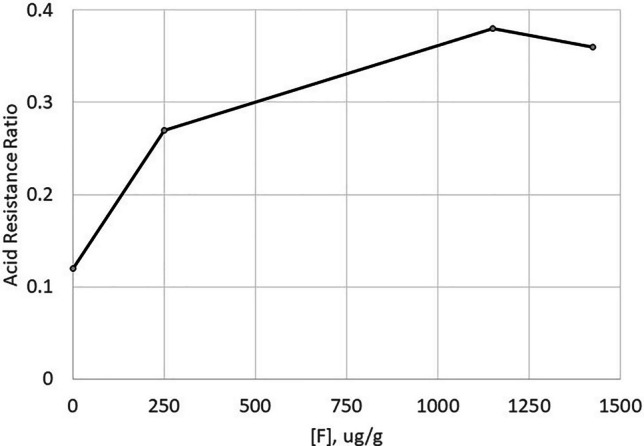
Reduction in demineralisation (measured by acid resistance ratio) as a function of fluoride concentration in the toothpaste treatment (combined F/non-SLS and F/SLS treatments)

In contrast, for demineralisation inhibition, the NMA showed the ARR increase to tail off to a plateau as the fluoride level increased above 1150 ppm F (Fig. 5). This was a significant non-linear fluoride effect (*p* = 0.029); the additional exploratory NMA model suggested demineralisation inhibition reached a plateau at 1007ppm.

The RER also reflected this non-linear pattern with similar means for 1426ppm (−30.1%) and 1150ppm (−30.4%) F toothpaste. A significant non-linear fluoride effect (*p* = 0.015) was observed in the additional exploratory NMA model suggesting RER reached a plateau at 1078ppm.

#### Fluoride speciation: free fluoride ion vs. monofluorophosphate

The treatment with sodium monofluorophosphate (MFP) as the main differentiating ingredient, MFP/SLS, gave lower remineralisation and lower demineralisation inhibition compared to the non-SLS and SLS-containing benchmarks with the same F concentration as fluoride ion (1400-1450ppm F). Specifically, for remineralisation, the mean difference between F/non-SLS and MFP/SLS was 8.9% (*p* < 0.001); for F/SLS vs. MFP/SLS, the mean difference was 6.4%, which achieved borderline significance (*p* = 0.065) despite not being included in the same study. Differences in demineralisation inhibition were not statistically significant (*p* > 0.4) but, of note, the ARR for F/non-SLS (PNP140) was higher than for MFP/SLS (CRB140) (mean difference = 0.08).

The other MFP-containing system, MFP/SLS/Zn/Cit, also gave low mean remineralisation and low mean inhibition of demineralisation. Only the F/Sn-Zn/SLS/LPP product had a lower mean remineralisation. However, the presence of Zn and citrate in this product means the specific contribution of MFP to the observed performance can’t be determined.

### Effect of Vehicle

#### Mouthwash versus, and in addition to, toothpaste

The mouthwash-only treatments means were ranked above any of the toothpaste-only treatments means (Fig. 2). The best-performing treatments from the NMA were the combination of fluoride toothpaste followed by use of a fluoride mouthwash (i.e. two fluoride doses). The single highest performing treatment was the highest-concentration fluoride F/non-SLS toothpaste followed by the highest-concentration fluoride mouthwash (PNP144): this was also the highest fluoride dose (6675 µg F).

Use of mouthwash in addition to fluoride toothpaste gave a significant increase (2.4%) in remineralisation versus fluoride toothpaste alone (*p* = 0.043). The 0.08 mean increase in demineralisation inhibition from using an additional mouthwash treatment was not statistically significant (*p* = 0.164).

#### Effect of other formulation ingredients on fluoride performance

The effects of formulation ingredients on fluoride performance were examined based on comparisons to the benchmark F/non-SLS ‘PNP’ product (Pronamel paste, as this was by far the most frequently tested fluoride system) at the relevant fluoride concentration.

For several formulations, the potentially impacting ingredient of interest was formulated into an SLS-containing base, so it was also useful to compare performance to the relevant ‘AQF’ product, as these also contained SLS, but no other potentially impacting ingredients. Although these comparisons had relatively low power (AQF formulations were included in only one study without any of the other products), treatment differences judged noteworthy are described below.

#### Effect of short-chain polyphosphates in toothpastes

The two polyphosphate formulations with short-chain polyphosphate as a potentially impacting ingredient (C3D110 and PPH110) both performed relatively poorly in this study. Of the fluoride-containing treatments, they were the overall lowest-ranked of the toothpastes with conventional F concentrations. For these products, both mean remineralisation and mean demineralisation inhibition were low, but only for remineralisation was the value statistically significantly lower than that of the relevant F/non-SLS benchmark (both *p* < 0.001). Of note, the ARR means for both polyphosphate products were 0.15 units lower than F/non-SLS (both *p* = 0.099).

#### Effect of Stannous-Zinc ion combination in toothpastes

The stannous fluoride products (F/Sn-Zn/SLS and F/Sn-Zn/SLS/LPP) showed a marked divergence from the other fluoride toothpastes in their behaviour in this in situ model, clearly evident from Fig. 2. Their remineralisation-promotion values were relatively very low, but their demineralisation-protection values were strong: indeed, the F/Sn-Zn/SLS/LPP toothpaste, containing long-chain polyphosphate in addition to stannous-zinc, gave the lowest remineralisation value and yet the highest demineralisation inhibition value of any of the toothpaste-only treatments.

Comparing F/Sn-Zn/SLS to F/non-SLS (i.e. CPS110 vs. PNP110, to avoid any confounding effect of LPP), the remineralisation value was much lower (delta-SMHR 8.8%; *p* = 0.001); while there was little evidence of a difference in demineralisation inhibition (delta ARR: 0.02 in favour of F/non-SLS, *p* = 0.831). Comparing it to F/SLS (AQF110, i.e., both SLS formulations), there was evidence that the stannous-zinc system reduced remineralisation (delta-SMHR: 6.6%; *p* = 0.053); but may increase demineralisation inhibition (delta-ARR = −0.08, *p* = 0.528; note not tested in the same study).

#### Effect of SLS in toothpastes

The effect of SLS was investigated via two comparisons. The most-tested of the comparisons was F/SLS/PO4/Carb versus F/non-SLS (4 studies included both products): here, the F/non-SLS gave statistically significantly more remineralisation (delta-SMHR 3.3%, *p* = 0.006). For the more specific SLS-focused comparison of F/non-SLS versus F/SLS, there was only weak evidence of higher remineralisation (delta-SMHR 2.39%, *p* = 0.382) and higher demineralisation inhibition (delta ARR = 0.10; *p* = 0.234) from the F/non-SLS (PNP) formulation, although there was low power for this comparison as the products were never tested in the same study.

#### Other ingredient comparisons

In all other planned comparisons, treatment differences did not reach statistical significance.

## Discussion

### Key learnings

Important observations from this NMA were that, in this in situ model: (i) there was a clear dose-response of fluoride in a toothpaste on both promotion of enamel remineralisation and inhibition of enamel demineralisation; (ii) the effect of fluoride concentration on promoting enamel remineralisation (between 0 and 1450 ppm F in a toothpaste vehicle) had a different profile to that for inhibiting demineralisation (linear vs. hyperbolic respectively); (iii) MFP toothpastes were less able to promote remineralisation and less able to inhibit demineralisation than benchmark toothpastes containing free fluoride ion; (iii) fluoride mouthwashes were effective vehicles to deliver fluoride for enamel benefits, and, when used after brushing with fluoride toothpaste, led to greater remineralisation than from brushing alone; (iv) a range of common toothpaste ingredients can inhibit fluoride-induced remineralisation, including polyvalent metal ions and polyphosphates, with some evidence for a modest effect of SLS; (v) polyvalent metal ions can add to the protective effects of fluoride against dietary acid exposure.

In practice, this in situ enamel erosion model is more precise in its measure of intra-oral enamel remineralisation (as SMHR) than of demineralisation resistance (as ARR – see ‘Assumptions and limitations’ section below). However, the ARR measure may be of particular value because it follows an extended period of post-treatment intra-oral remineralisation, so the surface is potentially extensively fluoridated in a biologically relevant manner. The RER measure is best understood as an overall efficacy measure combining effects seen in the remineralisation and post-remineralisation demineralisation phases [21], i.e. a summation of the SMHR and ARR measures. As SMHR and ARR are already key endpoints in this study, RER is not a focus of discussion here.

The present analysis confirms individual study findings that changes in mineralisation status of the enamel used in this in situ model are highly sensitive to fluoride concentration and treatment vehicle. This sensitivity may be heightened as the model uses only superficially demineralised bovine enamel substrate, with an immature pellicle. This provides a relatively clean, accessible enamel site of action with a tendency to remineralise in saliva.

The important observations of the study are now considered in turn, in terms of what they tell us about how fluoride can strengthen and protect enamel, and how these effects are modulated by how the fluoride is applied.

### Effect of Fluoride concentration and source

The degree of remineralisation due to fluoride ion increased in an essentially linear fashion from zero up to at least 1426 ppm F in the formulation chassis used: higher fluoride levels should exert still-stronger remineralisation effects. A linear profile was not observed for demineralisation inhibition, however: here, the effect of fluoride was non-linear, with a plateau beyond about 1000ppm F. These relationships were also observed in the caries version of this model [22].

This difference in the fluoride dose-response effects on remineralisation and demineralisation inhibition indicates that the acid resistance of an enamel surface is not just determined by the degree of fluoride-induced remineralisation. Neither does acid resistance seem to be determined merely by fluoride deposition onto enamel: Nehme et al. studied fluoride uptake and demineralisation inhibition after brushing as a function of time, and suggested fluoride uptake to enamel peaks much earlier than the acid resistance of the fluoridated surface [23].

The mechanism underpinning this observation requires further study. It is possible that demineralisation inhibition may be primarily a function of the fluoridation status of the more superficial, more readily accessible enamel surface, which may be saturated at modest fluoride concentrations (less than 1000ppm in a toothpaste). SMHR, on the other hand, may be affected by remineralisation down to the least accessible, inner zone of the erosive lesion, and so requires high concentration of fluoride ion to fully achieve it. This deeper remineralisation may therefore be adding strength but not acid resistance.

The nine studies comparing the effect of 1150ppm F in the non-SLS base vs. fluoride-free placebo (PNP110 vs. PNP000) showed the in situ model provided a highly reproducible (for a clinic-based study) estimate of fluoride effects on eroded enamel. The range of values for the effect of 1150ppm F (Fig. 3) varied by about a factor of two in SMHR (i.e. lowest 6%; highest 12%), and a factor of 3 in ARR (i.e. lowest 0.15, highest 0.47). All studies showed a highly significant effect of fluoride in all measures. This provided a sound platform for the other comparisons within the NMA, which incorporated a placebo value as a consistent ‘anchor’ across all 14 studies.

The relatively poor performance of the MFP-containing formulations here (F/MFP/SLS and F/MFP/SLS/Zn/Cit) can be understood as follows. The active form of fluoride in this in situ model is believed to be free fluoride ion: for MFP, this requires intra-oral hydrolysis of the parent ion by phosphatases. This hydrolysis may happen rapidly in plaque-coated surfaces prone to caries, as plaque is rich in phosphatase [24]. However, the relatively clean, plaque-free enamel surfaces used in this model – which are believed representative of eroded surfaces in vivo - offers little opportunity for hydrolysis. It is therefore likely that the great majority of MFP is lost through saliva dilution, expectoration and swallowing before it can fully act on the erosive lesion.

### Effect of Mouthwash

The individual studies did not consistently show a benefit on enamel remineralisation over-and-above brushing with fluoride toothpaste alone, but the NMA across the 3 mouthwash studies showed a statistically significant (if relatively modest) effect. The analysis also showed some evidence of a benefit on demineralisation inhibition from fluoride mouthwash on top of fluoride toothpaste (mean ARR difference 0.08 in favour of the combination of mouthwash and toothpaste, *p* = 0.16).

The effects of use of fluoride mouthwash alone (i.e. compared to non-fluoride toothpaste) were strong (Fig. 2). Intriguingly, the remineralisation values of pastes and mouthwashes were very similar: the driver of the higher ranking for mouthwash versus toothpaste was the (albeit non-significant) difference in the demineralisation inhibition values. Note all the 1450ppm toothpastes were ranked lower than the 225 ppm F mouthwash (used with non-fluoride toothpaste) despite delivering a similar dose of fluoride (2139 or 2175 vs. 2250 µg F respectively). Further study is required in this area to clarify whether there are differential effects of fluoride mouthwash versus fluoride toothpaste. Mouthwashes are high-water, low-viscosity, low-foaming formulations; they appear to make highly effective enamel fluoride delivery vehicles.

### Effects of formulation ingredients

Most of the formulations evaluated in this set of studies were marketed products (with many studies involving direct/head-head comparisons). Such studies have real-world meaning, as they represent actual choices for consumers regarding which product they use. Within the assumptions discussed, the products tested in this study can be characterised meaningfully (if not perfectly) in terms of their impacting ingredients in a straightforward, systematic manner, see Table 2. Given that context, the analysis provides valuable insight into the impact of specific ingredients (or ingredient classes) likely responsible for the differences in the performance of the products.

### Effect of polyvalent metal ions

Stannous and zinc are the only polyvalent metal ions commonly used as soluble salts in oral hygiene formulations, and have been shown capable of inhibiting fluoride uptake and/or remineralisation due to their ability to interact with negatively charged groups on enamel surfaces (primarily phosphate), both in vitro [25, 26] and in situ [15, 22, 27]. However, to potentially counteract this effect, their strong interaction with enamel may protect the surface from dietary acid attack. This has been reported for both stannous [28–31] and zinc [21].

In this study, the negative effects of Sn & Zn used in an 1100ppm fluoride toothpaste (CPS110) on remineralisation were dramatic: remineralisation was no greater than from the fluoride-free placebo. A contrasting positive effect on demineralisation inhibition was also evident, however, as the ARR value was as high as any of the other toothpastes.

A key question here is whether the overall effect of the stannous-zinc system is positive or negative on enamel protection. The RER calculation (which combines remineralisation and demineralisation effects) clearly indicates that the stannous-zinc system in CPS110 has an overall negative effect: its strong inhibition of demineralisation does not compensate for its reduction in remineralisation. This finding contrasts with several reports comparing stannous and sodium fluoride products (identical or closely related to those tested here) involving in situ models that use profilometric assessment of treatment effects [14, 28–30]. In these protocols, stannous-based formulations were found to outperform sodium fluoride-based formulations in terms of degree of surface loss.

These contrasting results appear to be due to the balance of remineralisation versus demineralisation that the different protocols involve. In the present study, the extended (and we believe physiologically relevant) period of uninterrupted post-treatment intra-oral remineralisation may allow extensive fluoridation. This is experimentally difficult to achieve in profilometry-based protocols, which require rapid surface loss.

This situation means that in the present protocol, during the post-remineralisation acid challenge, the surfaces treated with stannous-zinc toothpaste have fluoride, stannous and zinc ions bound to their surface, but have undergone limited remineralisation. The benchmark sodium fluoride product-treated surfaces, in contrast, have enamel surfaces that are relatively more highly remineralised and more intensively fluoridated [15]. Our analysis indicates that the acid resistance of these two types of surface is broadly equivalent.

### Effect of polyphosphates

Polyphosphates such as pyrophosphate can bind to calcium ions on enamel surfaces [32]. They have well-characterised anti-mineralisation effects, and so are used as anti-calculus agents [33]. They can inhibit fluoride uptake and/or remineralisation of enamel lesions in situ (caries lesions: [34]; erosive lesions: [19]). However, similarly to the metal ions, polyphosphates have been known for many years to be able to inhibit enamel demineralisation (due to caries at least), whether short-chain [35–38] or long-chain [39].

None of the remineralisation values for the three polyphosphate-containing products (F/SLS/PP, F/Sn-Zn/SLS/LPP and F/non-SLS/PP) was greater than that of the fluoride-free control, indicating potent inhibition of fluoride-induced mineralisation processes by polyphosphates.

However, for the short-chain polyphosphates at least, no suggestion of a boost to demineralisation inhibition was observed in the present analysis: indeed values were numerically (if not significantly) lower than non-polyphosphate benchmarks (F/SLS and F/non-SLS). Short-chain polyphosphates therefore seem unsuitable for toothpastes designed to reduce enamel erosion.

There was some evidence of a demineralisation inhibition benefit of long-chain polyphosphates: the ARR value of F/Sn-Zn/SLS/LPP was numerically higher than F/Sn-Zn/SLS and the highest of any toothpaste treatment, but further work is required to characterise this effect.

### Effect of SLS

SLS can inhibit fluoride uptake and fluoride-induced remineralisation [40, 41]. Highly anionic polymers such as polyacrylic acid used as thickening agents in toothpastes can inhibit demineralisation [42, 43], but they may also inhibit remineralisation due to their affinity for positively charged groups on enamel surfaces.

Statistically the most powerful comparison relevant to the effect of SLS in the present study was that of F/SLS/PO4/Carb versus F/non-SLS, which was tested in four head-head studies. The F/non-SLS product gave a modest but significantly superior remineralisation benefit (*p* = 0.006), without a detectable difference in demineralisation inhibition. It is likely that the principal formulation difference responsible for the lower remineralisation of the F/SLS/PO4/Carb formula was the inclusion of SLS, although a negative contribution from either the phosphate [44] or carbomer [42, 43] cannot be ruled out.

The other comparisons of +/- SLS products were from products not tested in the same study, so lacked the statistical power to add meaningful further evidence.

### Relation to potential evaluation in clinical studies

The formulation and delivery vehicle effects on in situ enamel remineralisation-demineralisation processes observed here can help direct formulation choice for future clinical studies. The primary focus, for daily-use oral care products as protection against erosive toothwear, would be the effect of fluoride salt and fluoride ion concentration (in the conventional range) in a toothpaste. This is because, although there is consensus fluoride ion is protective [8, 10–13], there remains some concern as to the extent, and to the impact of the counter-ion [30]. For the fluoride salt question, this should be a study comparing stannous fluoride with sodium fluoride (both well-formulated), at the same concentration, between 1000-1500ppm F. For the fluoride concentration question, a dose-response up to 1500ppm F, as sodium fluoride in a common silica base without polyvalent metal ions or polyphosphates, would seem appropriate. It would be important to compare against a fluoride-free (or low-fluoride) control, which would likely be a significant limitation to study duration (due to ethical considerations). A 5000ppm F positive control would also be valuable [45].

A key secondary focus would be to investigate the importance of base formulation to fluoride efficacy: the obvious test, from the data presented here, is the effect of adding polyphosphate to a sodium fluoride-silica toothpaste, again in the 1000ppm-1500ppm concentration range.

### Assumptions and limitations of the study

#### The in situ model and studies included in the analysis

As this is a relatively specialised field, there are few published in situ models to assess treatment effects on dental erosion processes. The fact that the models that do exist investigate different aspects of the remineralisation-demineralisation process, and so produce different patterns of results, favoured focus in the present meta-analysis on studies using a consistent protocol. The study set analysed is therefore restricted to those in situ studies of early enamel erosion using a common methodology, in practice all sponsored by Haleon (previously GSK Consumer Healthcare). This allowing clear characterisation of formulation effects on performance in the protocol, but inevitably restricts the generalisability of the findings.

However, a key strength of this NMA is that the in situ model used in the studies includes a meaningful period - 4 h - for remineralisation in the oral cavity after treatment, which allows significant fluoride-induced rehardening of the enamel to occur (when the treatment contains high levels of bioactive fluoride). The authors believe this to be clinically relevant: the protocol aims to represent a typical individual’s daily experience of having a meal (with an acidic component), followed by oral hygiene with fluoride products, followed by a substantial period without acid challenge before their next meal (again with an acid component). This was achieved via a sequence of standardised dietary acid challenge (causing demineralisation), treatment-induced remineralisation, then a second standardised challenge. Long-term effects are likely to be due to the sum of many such cycles. However, as the protocol involves only a single treatment, and effects were measured by surface microhardness changes, the effects of longer-term use cannot be directly estimated from these results.

Based on this work, to get a more accurate view of long-term efficacy, the protocol could potentially be modified while retaining the advantages of the in situ approach. This would require changes to the appliance used in the present studies, as it is not suitable for overnight wear. From the results of this study, overnight wear is likely critical for treatment-induced reparative remineralisation and development of a more acid-resistant enamel surface. Ideally, a modified model would also include a physical challenge to the test enamel surface, such as toothbrushing, as observed patterns of erosive toothwear are generally due to contact with other surfaces as well as acidic challenge. The model protocol should include means to distinguish the relative impacts of these acidic and physical challenges. A model was developed some years ago towards this purpose, and a small-scale study was published with promising results [46].

Finally, it should be noted that treatment differences in the remineralisation measure (SMHR) were more clearly resolved than for the demineralisation-inhibition measure (ARR). This is because the ARR calculation includes the E2 value, whereas the SMHR value does not. The E2 value is the most variable of the measures taken: as the final one in the sequence, it compounds the variability from all the preceding stages.

#### Ingredient assumptions

The analysis of ingredient effects assumed that most common toothpaste components have no meaningful effect on fluoride’s ability to remineralise enamel and protect it from demineralisation. This seems reasonable, as there is little scientific rationale to expect a meaningful interaction with fluoride ion, or with enamel, from humectants (most commonly glycerin and sorbitol), dental-grade silicas, flavour oils, sweeteners, colours, the opacifier titanium dioxide, potassium ions or nitrate ions, or gum thickeners. An exception to this is the gum thickener carbomer (polyacrylic acid), a heavily charged anionic polymer, which has been shown able to reduce enamel demineralisation [42, 43], so is characterised here as a potentially impacting ingredient. Certain formulations contained multiple potentially impacting ingredients, so determining the relative impact of each was not possible.

#### The NMA analysis and its limitations

The results obtained support the distinct choices with respect to the NMA modelling approach used to investigate these effects. The most fundamental was the decision to use individual patient-level data (IPD), rather than aggregated data, from reported results. The availability of IPD allowed the ARR (which was not derived or reported in all but the most recent studies) to be determined post-hoc for the earlier studies an included in this NMA, so this approach was preferred. The other factors considered were related to arm- or contrast-based modelling, fixed or random study effects and how to incorporate the subject-dependent data from inclusion of cross-over designs. All these choices are planned to be discussed and compared in a separate publication.

The NMA used here is well-suited to the exploratory hypothesis-generating objectives of the research, but is bounded by some limitations from the potential bias due to the lack of between-study evidence for many products, poor connectivity for some products (e.g., AQF) to more common products (i.e., PNP000 and PNP110), and high between-study variability, especially for the ARR parameter, leading to wide confidence intervals for some estimates.

Certain methods used by different research groups measure different aspects of the remineralisation-demineralisation process from others, and so in practice introduce too much variability for a meaningful analysis to be made from combined data. As this NMA assessed studies using a single protocol, its resulting clearer conclusions should better inform broader questions regarding the effects of daily-use fluoride treatments on dental erosion.

## Conclusions

Key findings from this NMA of 14 in situ studies were: (i) fluoride toothpastes promoted enamel remineralisation and inhibited demineralisation, acting in a dose-dependent manner; (ii) MFP was less effective than free F ion (as NaF); (iii) fluoride mouthwash used after a fluoride toothpaste can provide additional enamel benefits; (iv) polyvalent metal ions and short-chain polyphosphates can substantially reduce fluoride-induced enamel remineralisation (v) these metal ions also appear able to boost enamel acid resistance. However, in this model the overall benefit was no greater than that of fluoride alone, when delivered from a well-formulated toothpaste; (vi) SLS, an ingredient in nearly every toothpaste, may have some negative impact on intra-oral enamel remineralisation.

The high dependency of the remineralisation-demineralisation benefits of a fluoride toothpaste on other ingredients in the formulation, as seen in this study, indicates there is much to be gained by formulation optimisation.

Development of improved methodology with an enamel-loss endpoint is required to determine how these results are likely to translate to the real-life situation.

## Data Availability

Anonymized individual participant data and study documents can be requested for further research from www.clinicalstudydatarequest.com.
